# Rumen Microbiome Composition Is Altered in Sheep Divergent in Feed Efficiency

**DOI:** 10.3389/fmicb.2020.01981

**Published:** 2020-08-25

**Authors:** Steven McLoughlin, Charles Spillane, Noel Claffey, Paul E. Smith, Tommy O’Rourke, Michael G. Diskin, Sinéad M. Waters

**Affiliations:** ^1^Animal and Bioscience Research Department, Animal and Grassland Research and Innovation Centre, Teagasc, Athenry, Ireland; ^2^Genetics and Biotechnology Laboratory, Plant and AgriBiosciences Research Centre (PABC), Ryan Institute, National University of Ireland Galway, Galway, Ireland

**Keywords:** Greenhouse Gas, feed efficiency, sheep, ruminant, microbiome, metagenomics, 16S RNA

## Abstract

Rumen microbiome composition and functionality is linked to animal feed efficiency, particularly for bovine ruminants. To investigate this in sheep, we compared rumen bacterial and archaeal populations (and predicted metabolic processes) of sheep divergent for the feed efficiency trait feed conversion ratio (FCR). In our study 50 Texel cross Scottish Blackface (TXSB) ram lambs were selected from an original cohort of 200 lambs. From these, 26 were further selected for experimentation based on their extreme FCR (High Feed Efficiency, HFE = 13; Low Feed Efficiency, LFE = 13). Animals were fed a 95% concentrate diet *ad libitum* over 36 days. 16S rRNA amplicon sequencing was used to investigate the rumen bacterial and archaeal communities in the liquid and solid rumen fractions of sheep divergent for FCR. Weighted UniFrac distances separated HFE and LFE archaea communities from the liquid rumen fraction (Permanova, *P* < 0.05), with greater variation observed for the LFE cohort (Permdisp, *P* < 0.05). LFE animals exhibited greater Shannon and Simpson diversity indices, which was significant for the liquid rumen fraction (*P* < 0.05). *Methanobrevibacter olleyae* (in liquid and solid fractions) and *Methanobrevibacter millerae* (liquid fraction) were differentially abundant, and increased in the LFE cohort (*P.adj* < 0.05), while *Methanobrevibacter wolinii* (liquid fraction) was increased in the HFE cohort (*P.adj* < 0.05). This suggests that methanogenic archaea may be responsible for a potential loss of energy for the LFE cohort. Bacterial community composition (Permanova, *P >* 0.1) and diversity (*P* > 0.1) was not affected by the FCR phenotype. Only the genus *Prevotella 1* was differentially abundant between HFE and LFE cohorts. Although no major compositional shifts of bacterial populations were identified amongst the feed efficient cohorts (*FDR* > 0.05), correlation analysis identified putative drivers of feed efficiency with *Ruminococcaceae UCG-014* (liquid, *rho* = −0.53; solid, *rho* = −0.56) and *Olsenella* (solid, *rho* = −0.40) exhibiting significant negative association with FCR (*P* < 0.05). *Bifidobacterium* and *Megasphaera* showed significant positive correlations with ADG. Major cellulolytic bacteria *Fibrobacter* (liquid, *rho* = 0.43) and *Ruminococcus 1* (liquid, *rho* = 0.41; solid, *rho* = 41) correlated positively with FCR (*P* < 0.05). Our study provides evidence that feed efficiency in sheep is likely influenced by compositional changes to the archaeal community, and abundance changes of specific bacteria, rather than major overall shifts within the rumen microbiome.

## Introduction

The world’s population is expected to increase by 2 billion persons in the next 30 years, from 7.7 billion currently to 9.7 billion in 2050 (UN, 2019). In addition, rising gross domestic product (GDP) in developing countries and urbanization is driving dietary shifts toward animal-based protein products (Thornton, 2010; Henchion et al., 2017). There is increasing demand on livestock production systems to support the dietary requirements and demand of a rapidly growing population (Hunter et al., 2017). Feed is the largest economic factor influencing profitability in livestock enterprises, accounting for up to 70% of total direct costs (Kenny et al., 2018). Due to the cost of feed as an external input, improving profitability of livestock systems has significantly focused on the identification of animals capable of maximizing the utilization of feed (McGovern et al., 2018). Research to date provides evidence that highly feed efficient animals consume less feed, while at the same time maintaining the same level of production as less efficient animals (Carberry et al., 2012; Shabat et al., 2016; Claffey et al., 2018). Additionally, highly efficient animals produce less methane and less manure due to reduced consumption of feed (Kenny et al., 2018). Therefore, improving feed efficiency has the potential to simultaneously increase profitability within the livestock sector while reducing the environmental impact of livestock production.

Feed Conversion Ratio (FCR) and Residual Feed Intake (RFI) are two widely used measures of feed efficiency (Bhatt et al., 2013; Zhang et al., 2017; Claffey et al., 2018; McGovern et al., 2018). FCR is calculated as the kilogram ratio of dry matter intake (DMI) to average daily gain (ADG), while RFI measures the residual difference between observed and predicted feed intake against bodyweight maintenance and animal performance (Berry and Crowley, 2013). FCR and RFI have an inverse relationship with feed efficiency, with superior FCR and RFI measures corresponding to poorer animal production, and vice versa (Cannas et al., 2019). Both measures are related as they both require feed intake as a variable. However, a major limitation of FCR is that it is dependent on ADG, which can result in the selection of larger and faster growing animals that require more maintenance. In contrast, RFI is independent of growth rate and considered a more robust measurement of feed efficiency (Santana et al., 2012).

Ruminants depend on the microbes (composed mainly of bacteria, archaea, fungi, and protozoa) residing in the rumen to ferment and transform their feed into volatile fatty acids (VFAs), proteins and vitamins. The primary VFAs produced (acetate, propionate, and butyrate) contribute approximately 80% of the hosts metabolized energy requirements (Keogh et al., 2017; Li and Guan, 2017; Abecia et al., 2018; Zeineldin et al., 2018). Furthermore, the concentrations of different VFAs within the rumen have been associated with feed efficiency of the host (Li and Guan, 2017). The underlying biological mechanisms regulating production efficiency are dependent on a number of internal and external factors including age, sex, genotype, and diet, all of which are known to influence rumen microbial structure and function (Henderson et al., 2015; Shabat et al., 2016; Claffey et al., 2018; Thomas et al., 2019). Hence, there is a potential association between feed efficiency and the rumen microbiome. Indeed, previous research performed by our group and others has identified links between the rumen microbiome and animal variation in feed efficiency phenotypes (Carberry et al., 2012; Jewell et al., 2015; Shabat et al., 2016; Ellison et al., 2017; McGovern et al., 2018).

Understanding of microbiome composition and functioning has advanced in recent years through the application of high-throughput next-generation sequencing (NGS) technologies for metagenomic analyses (Quince et al., 2017). Popular NGS platforms such as the MISeq (Illumina) and MINion (Oxford Nanopore) coupled with metagenomic approaches that either target specific genes (16S rRNA) or the whole bacterial genome, are providing insights into complex microbial populations in the rumen, which are otherwise difficult to identify using culture-dependent approaches (Zhou et al., 2015; Kachiprath et al., 2018; Gu et al., 2019). Additionally, the development of user-friendly computational software is enabling researchers to extrapolate more information from biological data. For instance, CowPI, a functional prediction tool, can infer the functional potential of different rumen microbiome profiles using 16S rRNA data (Wilkinson et al., 2018).

There are approximately 1.2 billion sheep in the world that are primarily reared for commodities such as meat, milk and wool (Pulina et al., 2018). Sheep production remains an important agricultural enterprise internationally, which is exemplified by continual annual growth of the sheep dairy sector (Pulina et al., 2018). To date, most research investigating the relationship between feed efficiency and the rumen microbiome has been conducted in cattle. However, sheep are less expensive, require less feed, reach maturity quicker and are more manageable than cattle, making sheep a practical and economical model for ruminant research (Delano et al., 2002).

In a previous study by our group (Claffey et al., 2018), FCR was measured for a cohort of Texel cross Scottish Blackface (TXSB) lamb rams over 36 days and was found to vary across the group. While the rumen microbiome has been shown to be associated with feed efficiency in cattle (Carberry et al., 2012; Jewell et al., 2015; McGovern et al., 2018) such a relationship has not been extensively examined in sheep. Therefore, the objective of the current study was to investigate the bacterial and archaeal populations present in both solid and liquid fractions of the rumen of sheep that are divergent for the FCR phenotype, using amplicon sequencing targeting the 16S rRNA gene. In addition, the archaeal and bacterial populations identified were correlated with FCR to further identify possible microbial drivers of feed efficiency. To determine the potential functionality of the microbiome taxa that are differentially abundant due to FCR, CowPI (Wilkinson et al., 2018) was used to predict functional genes of metabolic pathways associated with feed efficiency in sheep.

## Materials and Methods

All animal procedures used in this study were conducted under experimental license from Ireland’s Health Products Regulatory Authority (HPRA) in accordance with the European Union (EU) protection of animals used for scientific purposes regulations 2012 (S.I. No. 543 of 2012). This study was conducted as part of a larger study designed to examine the production efficiency of purebred Scottish Blackface and TXSB wether and ram lambs (*n* = 200) (Claffey et al., 2018). The current study focused on the rams of the TXSB breeds of sheep used in that study. Briefly, twenty-six lambs of the TXSB were separated into divergent feed conversion efficiency quartile cohorts [high and low feed efficiency (HFE and LFE) animals with 13 animals in each group] according to their extreme FCR values, from an original group of 50 individuals [HFE = 3.83 ± 0.40, LFE = 6.05 ± 0.92, (*p* < 0.05)]. The experiment was performed over a period of 36 days of intensive indoor feeding. Lambs were individually penned on expanded metal-floored feeding pens (182 cm L × 122 cm W) and allowed tactile, olfactory, and visual contact with each other through the pen partitions. Lambs were allowed a 12-days pre-experimental acclimatization period to adapt to a 95% concentrate diet. Relative to commencement of *ad libitum*, concentrate feeding (day 0), lambs were offered 150-g/d fresh weight of concentrate feed on days −12, −11, and −10 increasing by 100-g/d fresh weight concentrate on each day from days −9 to day −1 to minimize the risk of any digestive upsets. For the duration of the finishing period, lambs were offered 100-g/d DM of silage and had *ad libitum* access to concentrates; *ad libitum* concentrate was described as access to concentrate feed at all times over the 36-days experimental period. Concentrate and silage samples were collected weekly and dried overnight at 55°C and pooled for determination of CP, ADF, NDF, and ash. Concentrate and silage were offered daily with individual lamb refusals recorded twice weekly (Claffey et al., 2018). Lambs were transported to the slaughter facility on the morning of slaughter. Animals were slaughtered at a mean age of 292 days old. Production variables [average daily feed intake (ADI), total weight gain (TWG), FCR, and ADG] were calculated post slaughter. All production data used in the study has been previously described (Claffey et al., 2018).

### Rumen Sampling, DNA Extraction and 16s rDNA Library Preparation

Liquid and solid fractions from rumen content were collected immediately after slaughter. Fractions were separated by squeezing rumen digesta through four layers of sterile cheesecloth, which were collected in 250 ml centrifuge bottles. Both fractions were frozen immediately in liquid nitrogen after separation and then stored at −80°C. Under liquid nitrogen, each sample was homogenized to a fine frozen powder using a pestle and mortar. Extraction of microbial DNA from the samples was performed using the method described by Yu and Morrison (2004). DNA purity was assessed using Nanodrop 1000 spectrophotometer. The 260/280 ratio averaged 1.83. To generate the PCR amplicons of the V4 hyper-variable region (of the 16S rDNA), 515F-806R primers were used on a template of 25 ng of rumen microbial DNA (Caporaso et al., 2011). 515F-806R primers target both bacterial and archaeal populations (Willis et al., 2019). The 515F-806R primers were designed with Nextera overhang adapters. The PCR amplification was conducted using 2X KAPA HiFi HotStart ReadyMix DNA polymerase (Roche Diagnostics, West Sussex, United Kingdom). The PCR conditions were as described in McGovern et al. (2018). Finally, the amplicons were sequenced on an Illumina MiSeq platform using the 500-cycle version 2 MiSeq reagent kit (Illumina, San Diego, CA, United States).

### Bioinformatic Analysis

Raw paired-end sequenced reads were quality checked with FASTQC (version 0.11.5) (Andrews, 2010). Primers and ambiguous basecalls were removed using Cutadapt (version 1.18) (Martin, 2011). Processing and analysis of amplicon reads was performed using Divisive Amplicon Denoising Algorithm 2 (DADA2), as described in Callahan et al. (2017). Read filtering, dereplication, sample inference, chimera removal, merging of paired end reads, and taxonomic classification were all performed following the DADA2 tutorial from https://benjjneb.github.io/dada2/tutorial.html (version 1.12) with minor alterations. Taxonomic classification was performed to the genus level using the SILVA classification database (sourced from https://zenodo.org/record/1172783#.XWLkpd-YW6A) (Callahan, 2018). The final output from DADA2 was an Amplicon Sequence Variant (ASV) table and a corresponding taxonomy table. A phylogenetic tree was constructed using the phangorn package (Schliep, 2011). A phyloseq object containing the ASV table, taxonomy table, phylogenetic tree and experimental metadata was built using the R/Bioconductor package Phyloseq (version 1.26) (McMurdie and Holmes, 2013) prior to downstream analysis. Finally, CowPI was used to predict the functional processes of the microbial community within each sample using the ASVs generated from the DADA2 pipeline (Wilkinson et al., 2018). Basic local alignment search tool (BLAST) against the rRNA/ITS database was used to further classify methanogens representative ASV sequences of interest (Johnson et al., 2008).

### Compositional and Statistical Analysis

Compositional and statistical analyses were carried out using various libraries/packages in R studio (running R version 3.6.1). Samples were separated according to rumen phase (liquid and solid) for independent analysis and compared between the feed efficient cohorts (LFE vs. HFE). Taxa unassigned at the phylum level, with less than 5 counts and prevalent in 3 or less samples were filtered from the data. For the analysis of alpha and beta diversity counts were normalized by subsampling to the minimum sampling depth; bacteria reads (liquid = 63,924, solid = 75,873) and archaea reads (liquid = 896, solid = 1182). Principle coordinate analysis (PCoA) based on weighted and unweighted UniFrac distances was performed for ordination analysis to visualize compositional differences between the two cohorts for both rumen fractions. PERMANOVA analysis with 9999 permutations was conducted using the Adonis function from the R/Bioconductor package Vegan (version 2.5-5) (Oksanen et al., 2019). Vegans betadisper and permutest functions were used to test for homogeneity of variance. Alpha diversity indices Shannon, Simpson, and observed ASVs were obtained for each of the rumen samples and compared between cohorts using the non-parametric Wilcoxon rank sum test. Alpha and beta diversity analysis was conducted at the ASV level for both bacterial and archaeal populations.

To profile the bacterial community populations, taxa were agglomerated to higher taxonomic ranks (i.e., phylum to genus) due to poor classification at the species level and counts were transformed to relative abundances. Archaeal populations were assessed at the genus and ASV level. Differential relative abundance analysis was conducted from phylum to genus level for bacteria populations and conducted at the genus to ASV level for archaea populations. For lower taxonomic ranks (i.e., genus and ASV) analysis was only conducted on taxa had a relative abundance greater than 0.1% and were prevalent at least 30% of samples. The Wilcoxon rank sum test was implemented to test for differences in relative abundance of taxa between the cohorts, and Benjamini-Hochberg (B-H) was used to correct for multiple testing. Spearman’s correlation analysis was also performed to test for associations between relative abundance of taxa and production traits of feed efficiency (FCR and ADG).

STAMP (v.2.1.3) (Parks et al., 2014) was used to conduct principal component and statistical analysis following functional prediction using CowPI (Wilkinson et al., 2018). The relative abundance of reads mapped to each functional process was compared between cohorts using Whites non-parametric *t*-test with B-H correction for multiple testing.

## Results

### Animal Production Traits Differed Across the Divergent Feed Efficiency Cohorts

A Wilcoxon rank sum test was performed to test the null hypothesis that production traits; FCR, ADG, average daily intake (ADI), TWG, did not differ between the two cohorts. For the four production traits significant differences were found in their medians (*p* < 0.05), confirming that production traits were statistically different between feed efficiency cohorts (Table 1; Claffey et al., 2018).

**TABLE 1:** Production traits related to feed efficiency (FCR, ADG, ADI, TWG) analyzed per feed efficiency cohort.

Production traits			
Production Traits	HFE (mean ± *SD*)	LFE (mean ± *SD*)	Wilcox. *P*-value
FCR	3.83 ± 0.40	6.05 ± 0.92	6.41E-10
ADG	0.47 ± 0.08	0.27 ± 0.04	9.43E-09
TWG	17.03 ± 2.79	9.70 ± 1.58	9.43E-09
ADI	1.79 ± 0.25	1.63 ± 0.38	4.50E-02

*A Wilcoxon rank sum test was used to test for significance. HFE n = 13, LFE n = 13.*

### Over 1600 Unique ASVs Identified in Both Rumen Fractions

Following data processing, quality filtering and chimera removal, and a total of 6,326,753 amplicon reads remained for analysis (solid phase = 3,061,130, liquid phase = 3,265,623). The average number of reads per sample in the liquid rumen phase was 125,600, and 117,735 in the solid rumen phase. 1691 uniquely identified ASVs were obtained from the reads in both rumen fractions. After prevalence filtering and removal of unclassified ASVs at the phylum level 560 and 513 ASV’s mapped to the kingdom bacteria, while 12 and 11 ASV’s mapped to the kingdom archaea for liquid and solid rumen fractions, respectively. Initial exploratory analysis using PCoA ordination based on weighted UniFrac distances detected two samples from the LFE cohort as outliers (Animal ID: 10707 and 10835) (Supplementary Figure S1). Further investigation revealed that in both fractions the genus *Prevotella 1* had a relative abundance of approximately 70%. The samples from both animals were considered highly biased and removed prior to downstream analysis.

### Effect of Rumen Fraction and FCR on Microbial Community Composition and Diversity

**FIGURE 1:**
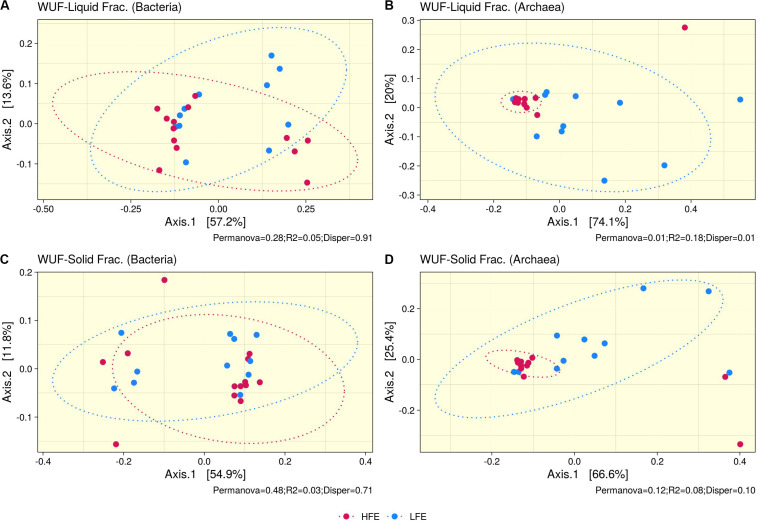
Beta diversity analysis. PCoA ordination plots based on weighted UniFrac distances for bacteria **(A,C)** and archaea **(B,D)** populations, for liquid **(A,B)** and solid **(C,D)** rumen fractions. Permanova *P*-value (Permanova), R2, and homogeneity of dispersion analysis (Disper) is provided for each analysis. Dots represent the different microbial samples and colors represent different feed efficient cohorts, HFE (Dark Pink) and LFE (Blue). HFE *n* = 13, LFE *n* = 11.

**FIGURE 2:**
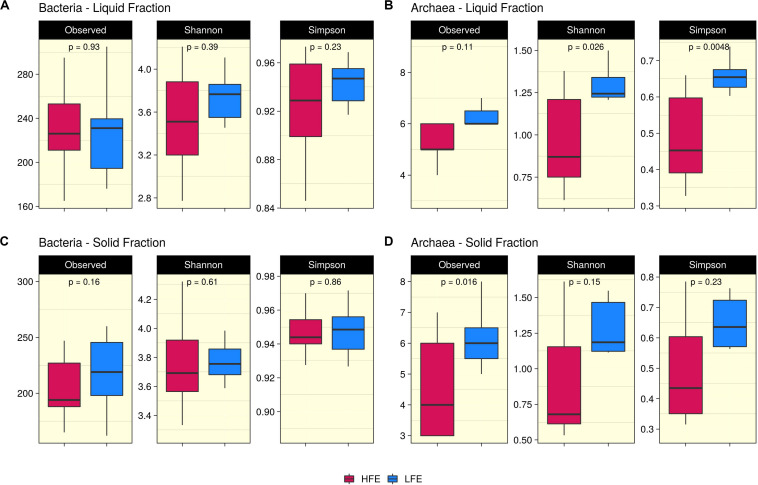
Alpha diversity analysis. Boxplots representing variations in alpha diversity in the rumen liquid **(A,B)** and solid **(C,D)** rumen fractions between high (Dark Pink) and low (Blue) feed efficiency cohorts. Alpha diversity metrics include Shannon, Simpson, and observed ASVs for both bacteria **(A,C)** and archaea **(B,D)** populations. HFE *n* = 13, LFE *n* = 11.

Ruminal fraction (i.e., liquid and solid) had no effect on microbial profiles (*P* < 0.05). Similar microbial composition, diversity and relative abundances were observed between the two fractions (Supplementary Figure S2). PCoA analysis on bacteria community composition showed considerable overlap between HFE and LFE samples, based on weighted (liquid, *P* = 0.28, *R2* = 0.05, *PermDisp* = 0.91; solid, *P* = 0.48, *R2* = 0.03, *PermDisp* = 0.71) (Figure 1) and unweighted (liquid, *P* = 0.10, *R2* = 0.06, *PermDisp* = 0.37; solid, *P* = 0.15, *R2* = 0.06, *PermDisp* = 0.69) UniFrac distances (Supplementary Figure S3). Alpha diversity indicators; Shannon, Simpson, and observed ASVs were not significant between HFE and LFE cohorts for either rumen fraction (*P* > 0.05), although LFE cohort exhibited greater diversity (Figure 2). For archaea populations greater variation in community composition was observed in the LFE cohort and found to be significantly divergent from the HFE cohort in the liquid rumen fraction based on weighted UniFrac distances (liquid, *P* = 0.01, *R2* = 0.18, *PermDisp* = 0.01; solid, *P* = 0.12, *R2* = 0.08, *PermDisp* = 0.10) (Figure 1). Shannon and Simpson indices were increased in the LFE liquid fraction (*P* > 0.05) and observed ASV was increased in the LFE solid fraction (*P* > 0.05) when compared to the HFE cohort (Figure 2).

### Significant Effects of FCR on Microbial Abundance

After filtering a total of 13 bacterial phyla were identified in both rumen fractions, however FCR phenotype did not affect their relative abundance. *Firmicutes* and *Bacteroidetes* constituted the most abundant phyla, respectively. Together they represented 77% and 83% of the mean relative abundance in the HFE cohort, and 82% and 83% of the mean relative abundance in the LFE cohort, for liquid and solid rumen fractions, respectively (Table 2). The *Firmicutes* and *Bacteroidetes* ratio (F:B) was not affected by FCR phenotype (*P* > 0.1) in either the liquid or solid rumen fractions. *Proteobacteria and Actinobacteria* were the next most abundant phyla, respectively. The mean relative abundance of *Fibrobacter* was increased in both HFE and LFE solid rumen fractions compared with liquid fractions, while also exhibiting a greater mean relative abundance in the LFE cohort compared the HFE cohort (*P* > 0.05) (Table 2).

**TABLE 2:** Mean relative abundance and standard deviation of bacterial phyla in rumen liquid phase for both HFE and LFE cohorts.

Rumen fraction	Phylum	HFE (mean)	HFE (*SD*)	LFE (mean)	LFE (*SD*)	Wilcoxon *P*-value	BH FDR	Spearman *rho* (FCR)	Spearman *P*-value
**Liquid**									
	F:B	0.67	0.25	0.69	0.20	1.00	NS	0.01	0.98
	Proteobacteria	0.18	0.13	0.12	0.11	0.07	0.30	–0.26	0.22
	Bacteroidetes	0.29	0.06	0.33	0.07	0.23	0.49	0.18	0.41
	Firmicutes	0.48	0.14	0.50	0.10	0.57	0.77	0.13	0.55
	Actinobacteria	0.04	0.04	0.02	0.01	0.57	0.77	–0.21	0.33
	Cyanobacteria	0.00	0.00	0.01	0.01	0.73	0.79	–0.07	0.73
	Fibrobacteres	0.00	0.00	0.01	0.02	0.09	0.30	0.44	0.03
	Spirochaetes	0.00	0.00	0.01	0.01	0.03	0.30	0.34	0.10
	Patescibacteria	0.00	0.00	0.00	0.01	0.65	0.77	–0.00	0.99
	Tenericutes	0.00	0.01	0.00	0.00	1.00	1.00	–0.33	0.12
	Synergistetes	0.00	0.00	0.00	0.00	0.27	0.50	0.38	0.06
	Kiritimatiellaeota	0.00	0.00	0.00	0.00	0.13	0.34	0.39	0.06
	Epsilonbacteraeota	0.00	0.00	0.00	0.00	0.60	0.77	–0.02	0.93
	Elusimicrobia	0.00	0.00	0.00	0.00	0.06	0.30	0.28	0.18
**Solid**									
	F:B	0.57	0.21	0.58	0.19	0.91	NS	0.18	0.39
	Proteobacteria	0.13	0.09	0.11	0.10	0.42	0.75	–0.06	0.79
	Bacteroidetes	0.28	0.07	0.29	0.04	0.91	0.91	0.19	0.38
	Firmicutes	0.54	0.12	0.54	0.13	0.91	0.91	–0.14	0.52
	Actinobacteria	0.03	0.02	0.01	0.01	0.12	0.39	–0.39	0.06
	Cyanobacteria	0.01	0.00	0.01	0.01	0.69	0.91	0.11	0.61
	Fibrobacteres	0.01	0.01	0.02	0.03	0.30	0.75	0.28	0.18
	Spirochaetes	0.01	0.01	0.01	0.02	0.11	0.39	0.28	0.19
	Patescibacteria	0.00	0.00	0.00	0.00	0.91	0.91	–0.10	0.65
	Tenericutes	0.01	0.01	0.00	0.00	0.36	0.75	–0.44	0.03
	Synergistetes	0.00	0.00	0.00	0.00	0.05	0.31	0.51	0.01
	Epsilonbacteraeota	0.00	0.00	0.00	0.00	0.72	0.91	–0.06	0.77
	Elusimicrobia	0.00	0.00	0.00	0.00	0.01	0.12	0.43	0.04
	Kiritimatiellaeota	0.00	0.00	0.00	0.00	0.46	0.75	0.13	0.54

*P-values are derived from Wilcoxon rank sum test and adjusted for false discovery rate using B-H method. Correlation coefficient derived using Spearman correlation to find associations between relative abundance and FCR. F:B denotes Firmicutes to Bacteroidetes ratio.*

**FIGURE 3:**
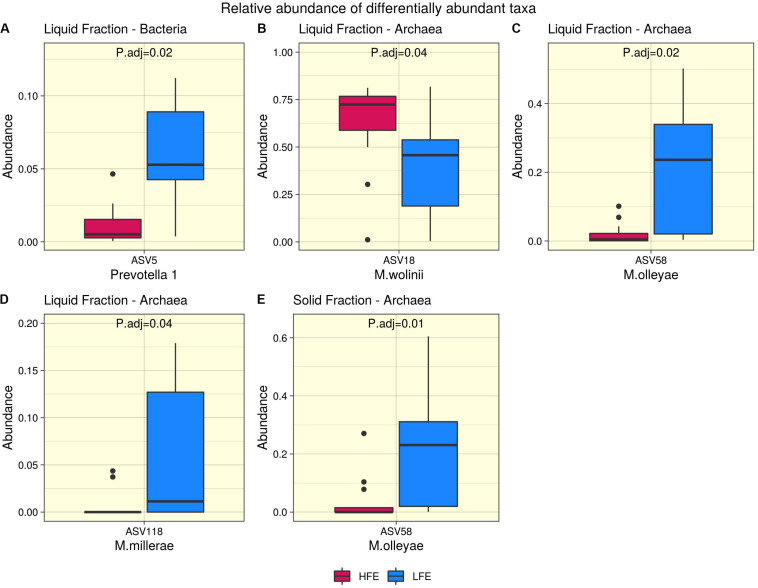
Differential abundance analysis. Bacteria **(A)** and archaea **(B–E)** taxa found to be differentially abundant between high (Dark Pink) and low (Blue) feed efficiency cohorts for liquid **(A–D)** and solid **(E)** rumen fractions. HFE *n* = 13, LFE *n* = 11.

**FIGURE 4:**
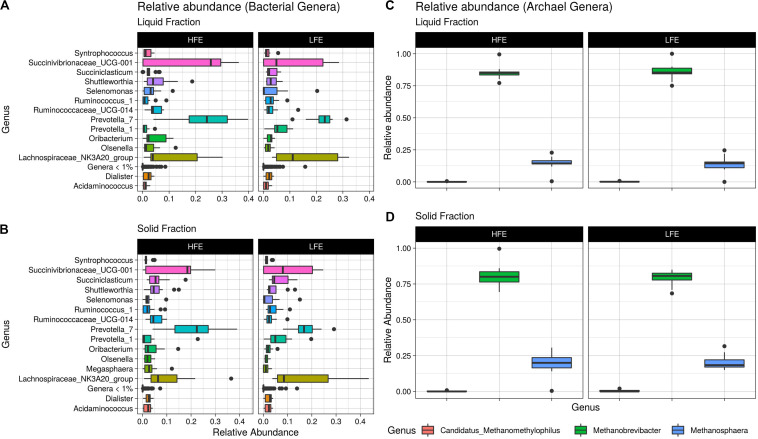
Relative abundance boxplots. Boxplots representing the variations in relative abundance of dominant bacterial genera (>1% relative abundance) **(A,B)** and archaea genera **(C,D)** in the rumen of HFE and LFE cohorts for liquid **(A,C)** and solid **(B,D)** rumen fractions. HFE *n* = 13, LFE *n* = 11.

A total of 104 and 99 bacterial genera were identified in the liquid and solid rumen fraction, respectively. The genus *Prevotella 1* was the only bacterial taxa differentially abundant, increased in the LFE liquid fraction (*FDR* = 0.02) (Figure 3). The most dominant genera in both fractions were *Prevotella 7*, *Succinivibrionaceae UCG-001* and *Lachnospiraceae NK3A20 group* (Figure 4) and their abundance did not differ between feed efficient cohorts (*P* > 0.05). Genera were predominantly enriched to families *Lachnospiraceae* (liquid = 24.0%; solid = 24.0%), *Ruminococcaceae* (liquid = 16.3%; solid = 17.0%), *Veillonellaceae* (liquid = 8.7%; solid = 9.0%), *Erysipelotrichaceae* (liquid = 8.7%; solid = 9.0%), and *Prevotellaceae* (liquid = 8.7%; solid = 9.0%).

After profiling of the archaeal population, three genera were identified. *Methanobrevibacter* was the most dominant, followed by *Methanosphaera* and *Candidatus Methanomethylophilus*, respectively, and their abundances were not affected by the FCR phenotype (*P* > 0.05). At the ASV level, 11 taxa were identified from the liquid rumen fraction and 10 from the solid rumen fraction. Three archaeal ASVs showed difference in relative abundance between the divergent FCR cohorts. In the liquid rumen fraction ASV58 and ASV118, identified to closely match *Methanobrevibacter olleyae* (98.8%) and *Methanobrevibacter millerae* (99.2%), respectively, were increased in the LFE cohort. Whereas ASV18, identified as *Methanobrevibacter wolinii* (100%) was increased in the HFE cohort. In the solid rumen fraction *M. olleyae* was increased in the LFE cohort (Figure 3).

### Significant Associations of Different Bacterial Taxa With FCR and ADG

Spearman’s correlation analysis was performed to identify putative bacterial drivers of feed efficiency. At the genus level, relationships were only explored for genera that were prevalent in greater than 30% of samples and had minimum relative abundance of 0.1%. At the genus level, in the rumen liquid phase the relative abundance of *Ruminococcaceae UCG-014* (*rho* = −0.51) exhibited the strongest negative correlation with FCR (*P* < 0.05), while *Prevotella 1* (*rho* = 0.56), *Coprococcus 1* (*rho* = 0.44), *Ruminococcus 1* (*rho* = 0.41), and *Fibrobacter* (*rho* = 0.43) exhibited the strongest positive correlation with FCR (*P* < 0.05). Only *Bifidobacterium (rho* = 0.41) exhibited a significant positive association with ADG (*P <* 0.05), while *Prevotella 1* (*rho* = −0.72), *Pseudoramibacter* (*rho* = −0.56), *Coprococcus 1* (*rho* = −0.51), *Ruminococcus 1* (*rho* = −0.50), *Ruminococcus 2* (*rho* = −0.43), *Acetitomaculum* (*rho* = −0.45), *Rikenellaceae RC9 gut group* (*rho* = −0.42), *Fibrobacter* (*rho* = −0.61), and *Treponema 2* (*rho* = −0.57) exhibited significant negative associations with ADG (*P* < 0.05) (Table 3).

**TABLE 3:** Spearman’s rank correlation showing dominant bacterial genera (>0.1%) that had a significant relationship with either FCR and/or ADG in the liquid rumen phase.

	Genus	HFE mean	HFE (*SD*)	LFE (mean)	LFE (*SD*)	*rho* (FCR)	*P*-value (FCR)	Rho (ADG)	*P*-value (ADG)
**Liquid**									
	Prevotella_1	0.01	0.01	0.06	0.04	0.56	0.01	–0.71	0.00
	Fibrobacter	0.00	0.01	0.02	0.02	0.43	0.03	–0.61	0.00
	Treponema_2	0.00	0.01	0.01	0.01	0.34	0.10	–0.57	0.00
	Pseudoramibacter	0.00	0.00	0.00	0.00	0.34	0.10	–0.56	0.01
	Coprococcus_1	0.00	0.00	0.00	0.01	0.43	0.03	–0.51	0.01
	Ruminococcus_1	0.02	0.03	0.03	0.03	0.41	0.05	–0.50	0.01
	Acetitomaculum	0.01	0.01	0.01	0.01	0.41	0.05	–0.45	0.03
	Ruminococcus_2	0.00	0.00	0.01	0.02	0.31	0.15	–0.43	0.04
	Rikenellaceae_RC9_gut_group	0.00	0.00	0.01	0.02	0.16	0.47	–0.42	0.04
	Bifidobacterium	0.01	0.01	0.00	0.00	–0.32	0.12	0.41	0.05
	Ruminococcaceae_UCG-014	0.05	0.03	0.03	0.04	–0.51	0.01	0.19	0.38
**Solid**									
	Prevotella_1	0.03	0.07	0.06	0.06	0.39	0.06	–0.56	0.01
	Ruminococcus_1	0.03	0.04	0.03	0.03	0.43	0.04	–0.53	0.01
	Pyramidobacter	0.00	0.00	0.00	0.00	0.53	0.01	–0.52	0.01
	Fibrobacter	0.01	0.02	0.01	0.03	0.28	0.18	–0.51	0.01
	Treponema_2	0.01	0.01	0.01	0.02	0.28	0.19	–0.48	0.02
	Roseburia	0.01	0.01	0.01	0.01	0.37	0.08	–0.45	0.03
	Pseudoramibacter	0.00	0.00	0.00	0.00	0.42	0.04	–0.45	0.03
	Bifidobacterium	0.00	0.00	0.01	0.00	–0.34	0.10	0.44	0.03
	Megasphaera	0.03	0.01	0.03	0.01	–0.20	0.36	0.43	0.04
	Acetitomaculum	0.01	0.02	0.01	0.01	0.42	0.04	–0.41	0.05
	Coprococcus_1	0.00	0.00	0.00	0.00	0.42	0.04	–0.41	0.05
	Olsenella	0.02	0.01	0.02	0.01	–0.40	0.05	0.33	0.12
	Ruminococcaceae_UCG-014	0.06	0.03	0.03	0.03	–0.55	0.01	0.29	0.17

*For analysis, only genera prevalent in more than 40% of samples were explored. HFE n = 13, LFE n = 11.*

In the solid rumen phase, *Ruminococcaceae UCG-014* (*rho* = −0.55) and *Olsenella* (*rho* = −0.40) exhibited a significant negative association with FCR (*P* < 0.05), while *Pyramidobacter* (*rho* = 0.53), *Pseudoramibacter* (*rho* = 0.42), *Ruminococcus 1* (*rho* = 0.43), *Acetitomaculum* (*rho* = −0.42), *Prevotella 1* (*rho* = 0.39), and *Coprococcus 1* (*rho* = 0.42) exhibited significant positive associations with FCR (*P* < 0.05). *Bifidobacterium* (*rho* = 0.44) and *Megasphaera* (*rho* = 0.43) were significantly positively associated with ADG (*P* < 0.05), while *Prevotella 1* (*rho* = −0.56), *Coprococcus 1* (*rho* = −0.41), *Ruminococcus 1* (*rho* = −0.53), and *Acetitomaculum* (*rho* = −0.41), *Roseburia* (*rho* = −0.45), *Pseudoramibacter* (*rho* = −0.45), *Fibrobacter* (*rho* = −0.51), *Pyramidobacter* (*rho* = −0.52), and *Treponema 2* (*rho* = −0.48) were among those showing significant negative associations with ADG (*P* < 0.05) (Table 3).

At the genus level, *Fibrobacter* (liquid, *rho* = 0.44), *Synergistetes* (liquid, *rho* = 0.038; solid, *rho* = 0.51), and *Elusimicrobia* (solid, *rho* = 0.43) exhibited a significant positive relationship with FCR (*P* < 0.05), whereas *Tenericutes* (solid, *rho* = −0.44) showed a significant negative relationship with FCR (*P* < 0.05) (Table 2).

### Functional Potential and Microbial Processes Similar Between Low and High Feed Efficient Cohorts

Principal component analysis, which captured over 75% variation with the first 2 principal components, indicated no separation in functional potential between feed efficient cohorts (Supplementary Figures S4, S5). No significant differences in metabolic processes were observed between cohorts for both liquid and solid rumen fractions (*P.adj* > 0.1).

## Discussion

The current study examined the effect of the FCR phenotype on ruminal bacteria and archaeal populations obtained from the liquid and solid rumen fractions of TxSB ram lambs offered a high concentrate diet *ad libitum.* Rumen liquid and solid fractions are widely used for microbiome research (De Mulder et al., 2016; Ji et al., 2017) and for animal feed efficiency analyses (McGovern et al., 2018). Both are differentiated ecological niches, which can contribute to feed efficiency in different ways. The solid rumen fraction is largely composed of plant and grain biomass and selects for various adherent cellulolytic and saccharolytic microbial species that play a significant role in the breakdown of feed within the rumen (De Mulder et al., 2016). While the liquid rumen fraction is considered to contribute less to the metabolic activity of rumen, it does provide readily available nutrients for free living microbes and facilitates their movement to newly consumed feed (De Mulder et al., 2016). Our analysis reveals a similar microbial community composition, diversity and relative abundance profiles between the liquid and solid rumen fractions (Supplementary Figure S2). A range of studies have also reported comparable findings in microbial diversity and community composition between liquid and solid rumen fractions in both cattle and sheep (Schären et al., 2017; McGovern et al., 2018; Li et al., 2020). The high degree of similarity observed between the fractions may be attributed to the method used to separate the fractions (McGovern et al., 2018). Alternatively, it may also reflect the frequent interchange of microbes between the liquid and solid fractions (De Mulder et al., 2016; Schären et al., 2017). Ultimately, due to the large degree of homogeneity observed between the two rumen fractions it is difficult to determine whether either plays a distinctive role toward feed efficiency in the current study.

In the rumen, methane production is considered beneficial to the host as it regulates the partial pressure of hydrogen facilitating microbial growth and promoting digestion within the rumen (Wallace et al., 2015; Lan and Yang, 2019). However, the production of methane is known to result in a loss of dietary energy to the host, of circa 2–12% depending on the diet (Johnson and Johnson, 1995), thereby impacting on the production performance of the animal. Several studies have linked higher methane emission to feed inefficiency in ruminants (Nkrumah et al., 2006; Zhou et al., 2010; Fitzsimons et al., 2013). The abundance of methanogenic archaea has also been correlated with higher levels of methane emissions (Wallace et al., 2015) and poorer feed efficiency (Zhou et al., 2010). As a result, numerous approaches have been developed to target rumen archaea to reduce methane emissions and improve animal production, including vaccines and small molecule enzyme inhibitors (Matthews et al., 2019). Although numerous studies in ruminants have reported no correlation between the overall abundance of methanogens and methane emissions, they have shown positive correlations between methane production and compositional changes within the archaea community (Danielsson et al., 2012; Shi et al., 2014; Tapio et al., 2017). In particular, increased abundance of taxa assigned to the *Methanobrevibacter* SGMT clade (i.e., *Methanobrevibacter gottschalkii, M. millerae*, and *Methanobrevibacter smithii)* is strongly correlated with methane emissions compared to those within the RO clade (i.e., *Methanobrevibacter ruminantium, M. olleyae, M. wolinii*) (Tapio et al., 2017). Members of the SGMT clade harbor 2 methyl coenzyme M reductase isozymes McrI and McrII, enabling them to utilize hydrogen more efficiently than those within the RO clade, which solely express McrI (Tapio et al., 2017). In line with such studies, our results show no major shifts in the relative abundance of archaea taxa at the genus level or higher taxonomic ranks between the feed efficient cohorts. However, compositional changes were observed at the ASV level. LFE animals exhibited greater variation in community composition (based on weighted UniFrac distances) (Figure 1) and increased diversity (as calculated by Shannon and Simpson and observed ASVs) (Figure 2) compared to their HFE counterparts. The relative abundance of *M. millerae* (SGMT clade) and *M. olleyae* (RO clade) was increased in the LFE liquid fraction, while *M. wolinii* (RO clade) was increased in the HFE liquid fraction. *M. olleyae* was also increased in the LFE solid fraction (Figure 3). Compositional changes within the *Methanobrevibacter* genus between divergent cohorts may partially explain the observed differences in feed conversion and animal production in our study.

Bacteria are the most diverse microbial domain found within the rumen and are capable of extracting energy from a wide variety of dietary substrates, including fiber, starch, sugars, and protein (Tapio et al., 2017). Due to the dependence of the host on bacterial fermentation it can be considered that the rumen bacterial population plays a critical role in the feed efficiency of the animal. Indeed, previous studies in both cattle and sheep have reported significant associations between the feed efficiency of the host and rumen bacterial populations (Jewell et al., 2015; Shabat et al., 2016; Ellison et al., 2017). In our study, no significant differences in bacterial alpha diversity between HFE and LFE lambs were observed (Figure 2). This is consistent with a number of studies in cattle (Myer et al., 2015; McGovern et al., 2018). Although, differences in alpha diversity were not significant in the present study, the HFE cohort exhibited a less diverse bacterial community than their LFE counterparts. This finding is in agreement with a larger study in cattle that reported lower bacterial diversity associated with higher feed efficiency (Shabat et al., 2016). Furthermore, we found no major shifts in community composition and relative abundance of taxa between feed efficient cohorts in either liquid or solid rumen fractions. Weighted UniFrac distances was unable to differentiate bacterial community composition between HFE and LFE cohorts with only a small percentage of the variation explained by the FCR phenotype (Figure 1). This finding was supported by our differential relative abundance analysis, which identified the genus *Prevotella 1* as the only bacterial taxa differentially abundant between the two feed efficient cohorts, increased in the LFE liquid fraction (Figure 3). While we detected no major differences in the relative abundance of taxa, we have identified several taxa exhibiting significant correlations of relative abundance with FCR and/or ADG (Table 3).

*Prevotella 1*, *Fibrobacter*, *Ruminococcus 1, Coprococcus*, *Pseudoramibacter*, and *Pyramidobacter* all exhibited significant positive associations with FCR and negative associations with ADG (Table 3). *Prevotella* species are known to ferment a wide variety of substrates including starches, peptides, proteins and hemicellulose (Matsui et al., 1998; Xie et al., 2019), which contribute to the feed efficiency of the host. Indeed, different *Prevotella* species have been associated with both higher and lower feed efficiency in cattle and sheep (Ellison et al., 2017; Brooke et al., 2019; Delgado et al., 2019). For instance, Ellison et al. (2017) reported that the abundance of *Prevotella ruminicola* increased significantly for L-RFI lambs when fed a concentrate based diet and decreased in L-RFI lambs when fed a forage-based diet. The opposite was reported for *Prevotella bryantii* (Ellison et al., 2017). This indicates a dietary effect on the abundance of *Prevotella* species, which is likely attributed to the metabolic divergence observed within the genus *Prevotella* (Matsui et al., 2000). Matsui et al. (2000) reported differential production of polysaccharide degrading enzymes and growth rates among *Prevotella* species when grown on various growth substrates *in vitro*. A recent study identified *Prevotella 1* as the most dominant genus in both the liquid and solid rumen fractions of lambs (Li et al., 2020). However, Li et al. (2020) offered lambs a higher ratio of forage to concentrate (45:55), which contrasts with the 95% concentrate diet provided to lambs in the current study. This suggests that *Prevotella 1* may require fibrous tissue or substrates released following fiber degradation for optimal growth, where the association with poorer feed efficiency in the current study may be driven by differences in the quantity of dietary intake observed between the two feed efficient cohorts (Table 1).

Members of the genera *Fibrobacter* and *Ruminococcus* are predominant fiber-digesting bacteria in the rumen, specifically the species *Fibrobacter succinogenes*, *Ruminococcus flavefaciens*, and *Ruminococcus albus* (Koike and Kobayashi, 2001). All of these three species largely depend on cellulose for growth and energy, although *R. albus* can utilize more efficiently a variety of other substrates produced following breakdown of plant fibers (La Reau and Suen, 2018). In contrast to our findings (Table 3), McGovern et al. (2018) found negative associations between RFI and the relative abundance of *Fibrobacter* and *Ruminococcus* OTUs. This may have resulted from variations in the ratio of dietary concentrates to forage fed to the animals during the two studies, in addition to differences in how feed efficiency was measured (FCR compared to RFI). Indeed, the abundance of these cellulolytic bacteria in the rumen has previously been shown to diminish with reductions in the ratio of dietary forages (Carberry et al., 2012; Henderson et al., 2015; Zhang et al., 2017). This suggests that the cellulolytic activities of *Fibrobacter* and *Ruminococcus* may become redundant in high concentrate-based diets, and that their increased abundance in the LFE cohort may confer inefficiency in energy extraction from feed.

Species within the genus *Coprococcus* metabolize carbohydrates for growth and energy, producing predominantly butyrate and acetate as fermentation end products (Whitman, 2015). In contrast to our findings (Table 3), previous studies in cattle have shown positive associations between *Coprococcus* and host feed efficiency (Jewell et al., 2015; McGovern et al., 2018). This may be explained by differences in animal models or diets used in the studies. Indeed, Kim et al. (2014) profiled the fecal microbiota from steers fed three different diets (high grain, moderate grain, and silage/forage) and found three distinct OTU’s belonging to *Coprococcus* that differed significantly between the treatment groups (Kim et al., 2014).

Associations between *Pseudoramibacter* and ruminant feed efficiency (Table 3) are not well reported in the literature. *Pseudoramibacter* is a member of the *Eubacteriaceae* family and can utilize carbohydrates for energy (Deusch et al., 2017), while producing fermentation end products butyrate, acetate, formate, and hydrogen (Deusch et al., 2017; Palakawong Na Ayudthaya et al., 2018).

The phylum *Synergistetes* was negatively correlated with feed efficiency in the solid rumen fraction (Table 2). This association was primarily driven by the genus *Pyramidobacter*. Members of the *Pyramidobacter* genus are asaccharolytic, non-motile and produce acetic acid and isovaleric acid (Downes et al., 2009). The abundance of *Pyramidobacter* has previously been associated with low RFI in Simmental bulls (McGovern et al., 2018) and isolated from higher methane emitting steers (Wallace et al., 2015). McGovern et al. (2018) found the relative abundance of *Pyramidobacter* and *Fibrobacter* to be positively correlated. This may indicate that *Pyramidobacter* relies on co-dependence with *Fibrobacter* for nutrient utilization following fiber degradation in the rumen.

The genera *Roseburia*, *Treponema 2*, *Mogibacterium, Rikenellaceae RC9 gut group*, *Acetitomaculum* and *Ruminococcus 2* all exhibited significant negative associations with ADG in either or both rumen fractions in the current study, but showed no significant associations with FCR (Table 3). *Roseburia* utilizes carbohydrates for growth and its abundance is known to increase with greater ratios of dietary concentrates (McCann et al., 2014; Zhang et al., 2018). Butyrate is the primary VFA produced by *Roseburia* and its production is largely dependent on the availability of acetate (Duncan et al., 2002). Supporting the findings of our study, Li et al. (2019) identified a greater abundance of *Roseburia* in feed inefficient Kinsella composite hybrid steers fed a high-energy diet (Li et al., 2019). In contrast, Ellison et al. (2017) reported a greater abundance of *Roseburia* in feed efficient lambs fed a concentrate diet. Other studies in this area have not reported any association between *Roseburia* and feed efficiency (Carberry et al., 2012; Jewell et al., 2015; McGovern et al., 2018). Given the saccharolytic activity of *Roseburia*, it is unclear why the genus correlated negatively with feed efficiency in lambs fed a high concentrate diet. One suggestion is that a greater availability of acetate may be present in the rumen of lower feed efficient lambs. *Roseburia* has been reported to be a net utilizer of acetate during growth (Duncan et al., 2002). The correlation between the abundance of *Acetitomaculum* and lower feed efficiency may support this possibility. The genus *Acetitomaculum* is capable of utilizing hydrogen to reduce carbon dioxide for the formation of acetate in a process known as acetogenesis (Greening and Leedle, 1989; Le Van et al., 1998).

*Mogibacterium* has previously been identified in the rumen of both sheep (Mi et al., 2018) and cattle (Myer et al., 2015; Freetly et al., 2020). *Mogibacterium* belongs to the order *Clostridiales* from the phylum *Firmicutes* and is described as incapable of breaking down carbohydrates for energy (Whitman, 2015). Similar to the finding presented in the current study (Table 3), a recent study found *Mogibacterium* to be enriched in the jejunum of lower ADG steers fed a high-energy diet (Freetly et al., 2020). In addition, *Mogibacterium* has also been associated with higher methane-emitting steers (Wallace et al., 2015).

Members of the genus *Treponema* have been associated with pathological conditions including digital dermatitis (Wilson-Welder et al., 2015), yaws disease and syphilis (Newbrook et al., 2017), while others are part of the normal microflora in the GI tract of animals (Newbrook et al., 2017). *Treponema* species such as *Treponema bryantii* and *Treponema succinifaciens* ferment carbohydrates (Cwyk and Canale-Parola, 1979; Stanton, 1984), but are also known to be involved in the breakdown of fiber (Xie et al., 2018). In a recent study (Ellison et al., 2019) identified a greater abundance of the species *Treponema maltophilum* in the rumen of feed efficient lambs fed a forage-based diet. McGovern et al. (2018) also identified two *Treponema* OTU’s positively correlating with feed efficiency in Simmental bulls. This contrasts with the findings of our study, which found *Treponema* associating significantly with poorer ADG and tending toward feed inefficiency in lambs fed a high concentrate diet (Table 3).

*Rikenellaceae RC9 gut group* has previously been identified in the rumen of domesticated livestock (Petri et al., 2013; Ishaq et al., 2019; Ren et al., 2019). Petri et al. (2013) observed a reduction in the abundance of *Rikenellaceae* in heifers treated with a diet comprising mixed forage and concentrate to those fed forage alone (Petri et al., 2013). Additionally, the abundance of unclassified *Rikenellaceae* was found to decrease in goats fed with high grain diets compared to hay based diets (Liu et al., 2015). The finding from these studies may indicate a preference for forage-based diets for the *Rikenellaceae RC9 gut group* and could explain its correlation with reduced ADG in our study where animals were fed a concentrate diet.

*Ruminococcaceae UCG-014* and *Olsenella* also exhibited significant negative associations with FCR and were not found to be significantly associated with ADG (Table 3). The uncultivable genus *Ruminococcaceae UCG-014* (family *Ruminococcaceae*) showed the strongest negative associations with FCR (Table 3). *Ruminococcaceae* is considered a dominant family within the rumen of livestock (Creevey et al., 2014; Henderson et al., 2015) and generally more abundant in animals fed forage-based diets (Henderson et al., 2015). Within the *Ruminococcaceae* family certain members are known cellulolytic fermenters, such as *R. albus* and *R. flavefaciens* (Perea et al., 2017). However, other members are non-cellulolytic and actively ferment various forms of polysaccharides (Hook et al., 2011; Petri et al., 2012; La Reau and Suen, 2018). Indeed, Ellison et al. (2017) found particular *Ruminococcus* species to be more enriched in sheep fed a concentrate diet, compared to those fed a forage-based diet and vice versa. Additionally, in a study conducted on dairy cows the abundance of *Ruminococcaceae NK4A214* was increased in a high grain diet (Pan et al., 2017). It is unclear why *Ruminococcaceae UCG-014* shows significant associations with FCR in our study. One possibility is that it may indicate that *Ruminococcaceae UCG-014* is associated with improved carbohydrate metabolism in the rumen.

*Olsenella* ferment starch and glycogen substrates and produce lactic, acetic, and formic acid (Göker et al., 2010). Members of the genus *Olsenella* have been identified in oral cavities and GIT of humans and animals (Kraatz et al., 2011; Ellison et al., 2017; Kubasova et al., 2018; Elolimy et al., 2020). In line with our findings (Table 3), Elolimy et al. (2020) reported a greater abundance of *Olsenella* in hindguts of feed efficient Holstein heifer calves (Elolimy et al., 2020). However, other studies have reported a greater abundance of *Olsenella* in the rumen microbiota of low feed efficient lambs when fed a concentrate diet (Ellison et al., 2017) and in the fecal microbiota of low feed efficient piglets (Kubasova et al., 2018).

The genera *Megasphaera* and *Bifidobacteria* exhibited significant positive associations with ADG but no significant associations with FCR (Table 3). *Megasphaera* has previously been associated with high feed efficiency in Holstein dairy cattle (Shabat et al., 2016), and also exhibited greater abundance in lower methane emitting sheep (Kamke et al., 2016). *Megasphaera* is known to metabolize lactate within the rumen, which it utilizes for production of important VFAs for animal growth (e.g., acetate, propionate, and butyrate) (Chen et al., 2019), indicating its association with ADG in ruminants. Removal of lactate is important mechanism in regulating pH levels within the rumen, preventing rumen lactic acidosis and maintaining rumen health and function (Hernández et al., 2014; Chen et al., 2019).

*Bifidobacterium* species are known to produce a broad spectrum of carbohydrate modifying enzymes, which facilitate the metabolism of a wide variety of dietary carbohydrates. This enables members of the *Bifidobacterium* genus to efficiently adapt, extract energy and contribute to the feed efficiency of the host when offered a high-energy diet (Pokusaeva et al., 2011). Indeed, Ellison et al. (2017) found *Bifidobacterium* to be significantly more abundant in the rumen of feed efficient lambs when fed a concentrate diet. Furthermore, a study conducted by Abe et al. (1995) showed that oral administration of *Bifidobacterium* improved daily weight gain and FCR of young calves (Abe et al., 1995). Our findings are consistent with both of these studies, suggesting that *Bifidobacterium* may contribute significantly in extracting energy from carbohydrate based diets.

The relationship of feed efficiency with the rumen microbiota composition and abundance has not been extensively researched in sheep. In one study, Ellison et al. (2017) examined the effect of feed efficiency, diet and breed on the rumen microbial populations from the rumen of sheep. That study differed from our study in several key aspects. Firstly, in the study by Ellison et al. (2017) RFI was used to distinguish feed efficient cohorts, which contrasts with the FCR measurement used in our study. Secondly, Ellison et al. (2017) used wether lambs spanning three different breeds of sheep – Rambouillet, Hampshire, and Suffolk, whereas TXSB ram lambs were used in our study. Thirdly, Ellison et al. (2017) fed animals with both concentrate and forage based diets. Although animals were not fed with a forage-based diet in this study, the composition of concentrates in the diets used in both studies varied. A further study carried out by Perea et al. (2017) also examined the effect of RFI on the microbial populations in the rumen, as well as multiple other sites from the digestive tract, of wether lambs fed a forage-based diet. Differences in diet, breeds, sex, measures of feed efficiency and analytical methodologies used between the studies may have contributed to the differences in the findings between the studies.

The dominance of *Firmicutes* and *Bacteroidetes* in the rumen of ruminants is widely reported throughout the literature (McGovern et al., 2018; Paz et al., 2018; Trabi et al., 2019; Liu et al., 2019). Consistent with those studies *Firmicutes* and *Bacteroidetes* were identified as the most abundant phyla in the rumen of ram lambs fed a high concentrate diet. *Prevotella 7*, *Succinivibrionaceae UCG-001*, and *Lachnospiraceae NK3A20* group were found to be the three most abundant genera in our study (Figure 3). This finding is also in line with a large global study set out to characterize the core rumen microbiota in small and large ruminants (Henderson et al., 2015). Henderson et al. (2015) identified *Prevotella* and unclassified *Lachnospiraceae* among the most abundant bacterial groups in the rumen. In addition, *Prevotella* and unclassified *Succinivibrionaceae* were found to be the most abundant bacterial groups in ruminants when fed a concentrate diet (Henderson et al., 2015). The degree of similarity between the taxa of sheep and cattle indicate that sheep models may serve as a useful and robust model for rumen microbiome research, as they are less expensive and more manageable than cattle (Delano et al., 2002).

To limit global warming to below 1.5°C above pre-industrial levels by 2050, in line with the Paris Agreement (United Nations/Framework Convention on Climate Change [UNFCCC], 2015), as well as feeding a growing population, there is an urgent requirement to increase production while reducing methane emissions intensity from livestock (Islam and Lee, 2019). In 2018, the IPCC’s Special Report on Global Warming of 1.5°C detailed reduction targets for global biogenic methane to between 24 and 47% of 2010 levels by 2050 (IPCC, 2018). Reducing methane emissions from livestock production represents a promising mitigation strategy that can be achieved by sustainable intensification of livestock production and/or reduced livestock product consumption (Herrero et al., 2016). In the context of sustainable intensification of ruminant livestock production, our findings indicate that genetically selecting for feed efficient animals can be a potential route for improving production and reducing feeding costs, while achieving methane emissions reductions in ruminant production systems.

In summary, our study investigated the rumen bacterial and archaeal populations in the rumen of ram lambs divergent for the FCR phenotype, which were fed a concentrate diet. Although lambs were found to be significantly divergent for feed efficiency, no major shifts in the rumen bacterial composition were observed. Correlation analysis suggests that the differences in feed efficiency may be attributed to a number of specific bacterial taxa within the rumen rather than the community as a whole. On the other hand, archaea community composition, diversity and the relative abundance of a *Methanobrevibacter* ASV differed between HFE and LFE cohorts, which may partially explain a loss of energy in the LFE cohort. Whilst no significant difference in predicted metabolic processes was detected using CowPI, a limitation of this technique is that it infers microbial metabolic pathways based of 16S rDNA data and does not measure the microbial transcriptome or proteome (Wilkinson et al., 2018). Our study was also somewhat limited by poor classification at the species level. The aforementioned limitations can potentially be overcome by the use of both shotgun metagenomics for greater resolution of taxonomic classification (Quince et al., 2017; Brumfield et al., 2020) and metatranscriptomics uncovering the functional potential of the rumen microbiome (Shakya et al., 2019). Our current study focused exclusively on interrogating bacterial and archaeal populations from the rumen. A more comprehensive understanding of the contribution of the sheep rumen microbiota to animal feed efficiency would ideally investigate all major microbial populations within the rumen, including protozoa and fungi (Newbold et al., 2015; Tapio et al., 2017).

## Data Availability Statement

The datasets presented in this study can be found in online repositories. The names of the repository/repositories and accession number(s) can be found below: https://www.ncbi.nlm.nih.gov/, PRJNA643304.

## Ethics Statement

The animal study was reviewed and approved by the Ireland’s Health Products Regulatory Authority (HPRA) in accordance with the European Union (EU) protection of animals used for scientific purposes regulations 2012 (S.I. No 543 of 2012). Written informed consent was obtained from the owners for the participation of their animals in this study.

## Author Contributions

MD and NC designed and conceptualized the animal model and collected rumen samples for analysis. SW, CS, and SM designed and conceptualized the microbiome study, carried out detailed interpretation of the data, and drafted the manuscript. TO’R and SW performed the DNA extraction and amplicon library generation. SW and PS coordinated the sequencing runs. SM conducted all of the bioinformatics and statistical analyses. PS provided assistance with data analysis. SM wrote the first draft of the manuscript, which was revised by SW and CS. All authors contributed to the final manuscript revision, read and approved the final draft of the manuscript.

## Conflict of Interest

The authors declare that the research was conducted in the absence of any commercial or financial relationships that could be construed as a potential conflict of interest.

## Supplementary Material

FIGURE S1 Beta diversity analysis. NMDS ordination plot based weighted and unweighted UniFrac distances. Dots represent the different samples. Colors represent different feed efficient cohorts, HFE (Pink) and LFE (Blue). The plots show outlying samples (10707, 10835), which were removed prior to downstream analysis. HFE *n* = 13, LFE *n* = 13. **(A)** Weighted Unifrac distances for liquid fraction (Bacteria). **(B)** Unweighted Unifrac distances for liquid fraction (Bacteria). **(C)** Weighted Unifrac distances for solid fraction (Bacteria). **(D)** Unweighted Unifrac distances for solid fraction (Bacteria).

FIGURE S2 Analysis of ruminal fractions. PCoA ordination plots with weighted **(A)** and unweighted **(B)** UniFrac distances. Alpha diversity analysis; Shannon simpson and observed ASV for solid and liquid rumen fractions **(C)**. Colors represent different feed efficient cohorts, HFE (Dark Pink) and LFE (Blue). Stacked bar chart representing the relative abundance of 10 most abundant genera **(D)**. HFE *n* = 13, LFE *n* = 11.

FIGURE S3 Beta diversity analysis. PCoA ordination plots based on weighted UniFrac distances for bacteria **(A,C)** and archaea **(B,D)** populations, for liquid **(A,B)** and solid **(C,D)** rumen fractions. Permanova *P*-value (Permanova), R2, and homogeneity of dispersion analysis (Disper) is provided for each analysis. Dots represent the different microbial samples and colors represent different feed efficient cohorts, HFE (Dark Pink) and LFE (Blue). HFE *n* = 13, LFE *n* = 11.

FIGURE S4 PCA plot comparing microbiome functional profiles for each sample sampled from the liquid rumen phase. HFE *n* = 13, LFE *n* = 11.

FIGURE S5 PCA plot comparing microbiome functional profiles for each sample sampled from the solid rumen phase. HFE *n* = 13, LFE *n* = 11.
