# Supplementary figures and images for: Rumen Microbiome Composition Is Altered in Sheep Divergent in Feed Efficiency
Source: Front Microbiol. 2020 Aug 25;11:1981. doi: 10.3389/fmicb.2020.01981 (PMC7477290; doi:10.3389/fmicb.2020.01981)

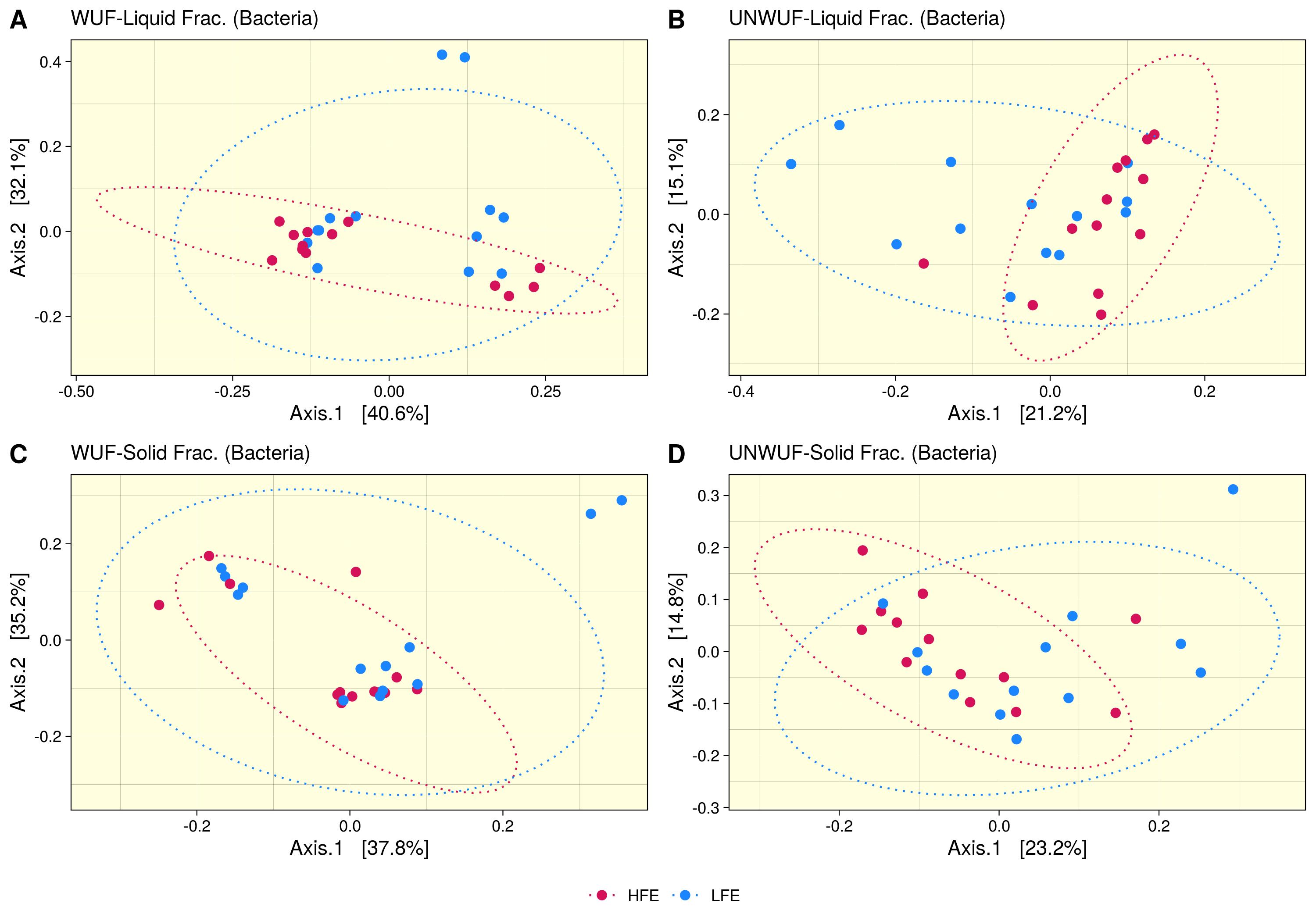

Supplement: FIGURE S1 — Beta diversity analysis. NMDS ordination plot based weighted and unweighted UniFrac distances. Dots represent the different samples. Colors represent different feed efficient cohorts, HFE (Pink) and LFE (Blue). The plots show outlying samples (10707, 10835), which were removed prior to downstream analysis. HFE n = 13, LFE n = 13. (A) Weighted Unifrac distances for liquid fraction (Bacteria). (B) Unweighted Unifrac distances for liquid fraction (Bacteria). (C) Weighted Unifrac distances for solid fraction (Bacteria). (D) Unweighted Unifrac distances for solid fraction (Bacteria). [file Image_1.JPEG]

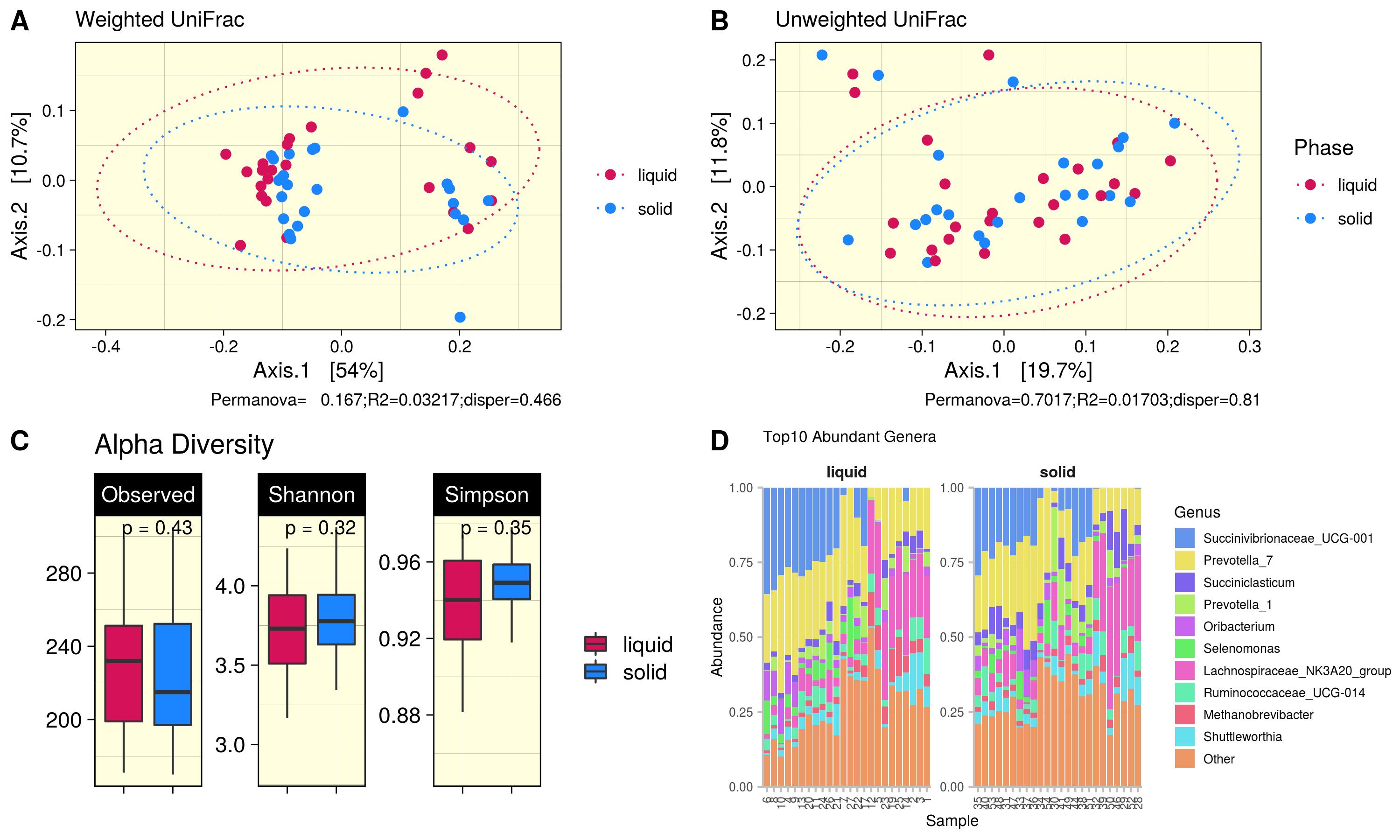

Supplement: FIGURE S2 — Analysis of ruminal fractions. PCoA ordination plots with weighted (A) and unweighted (B) UniFrac distances. Alpha diversity analysis; Shannon simpson and observed ASV for solid and liquid rumen fractions (C). Colors represent different feed efficient cohorts, HFE (Dark Pink) and LFE (Blue). Stacked bar chart representing the relative abundance of 10 most abundant genera (D). HFE n = 13, LFE n = 11. [file Image_2.JPEG]

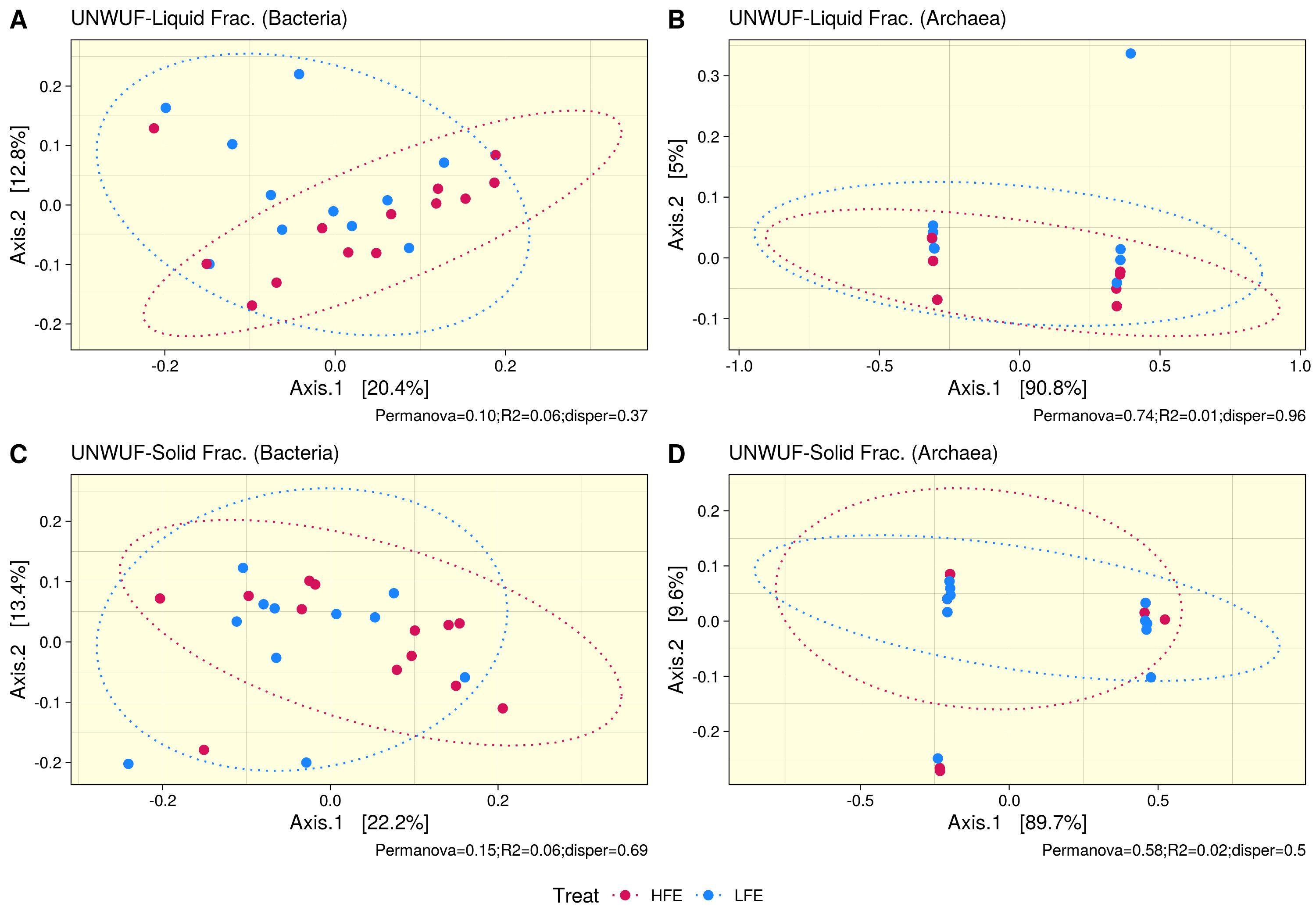

Supplement: FIGURE S3 — Beta diversity analysis. PCoA ordination plots based on weighted UniFrac distances for bacteria (A,C) and archaea (B,D) populations, for liquid (A,B) and solid (C,D) rumen fractions. Permanova P-value (Permanova), R2, and homogeneity of dispersion analysis (Disper) is provided for each analysis. Dots represent the different microbial samples and colors represent different feed efficient cohorts, HFE (Dark Pink) and LFE (Blue). HFE n = 13, LFE n = 11. [file Image_3.JPEG]

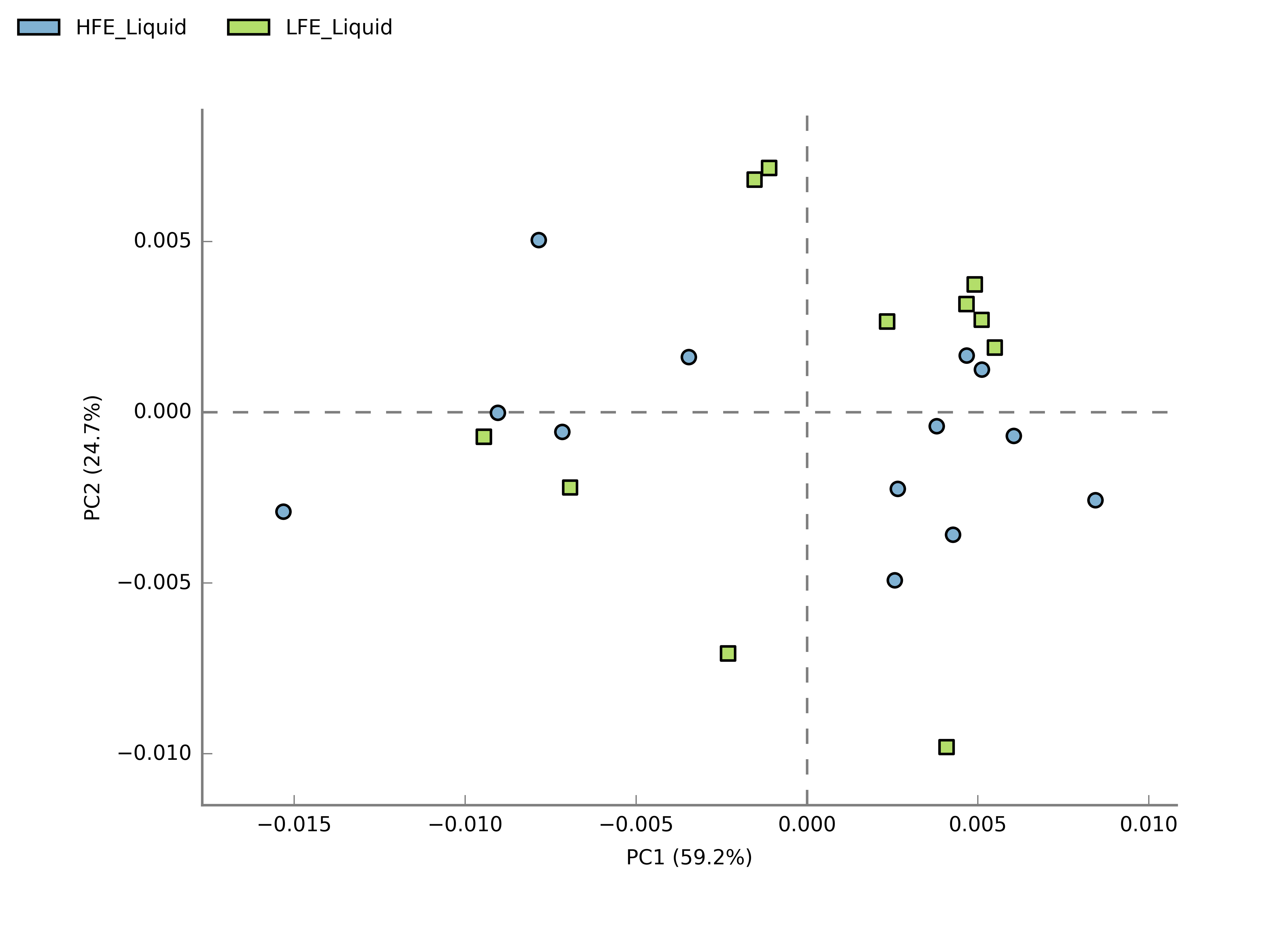

Supplement: FIGURE S4 — PCA plot comparing microbiome functional profiles for each sample sampled from the liquid rumen phase. HFE n = 13, LFE n = 11. [file Image_4.JPEG]

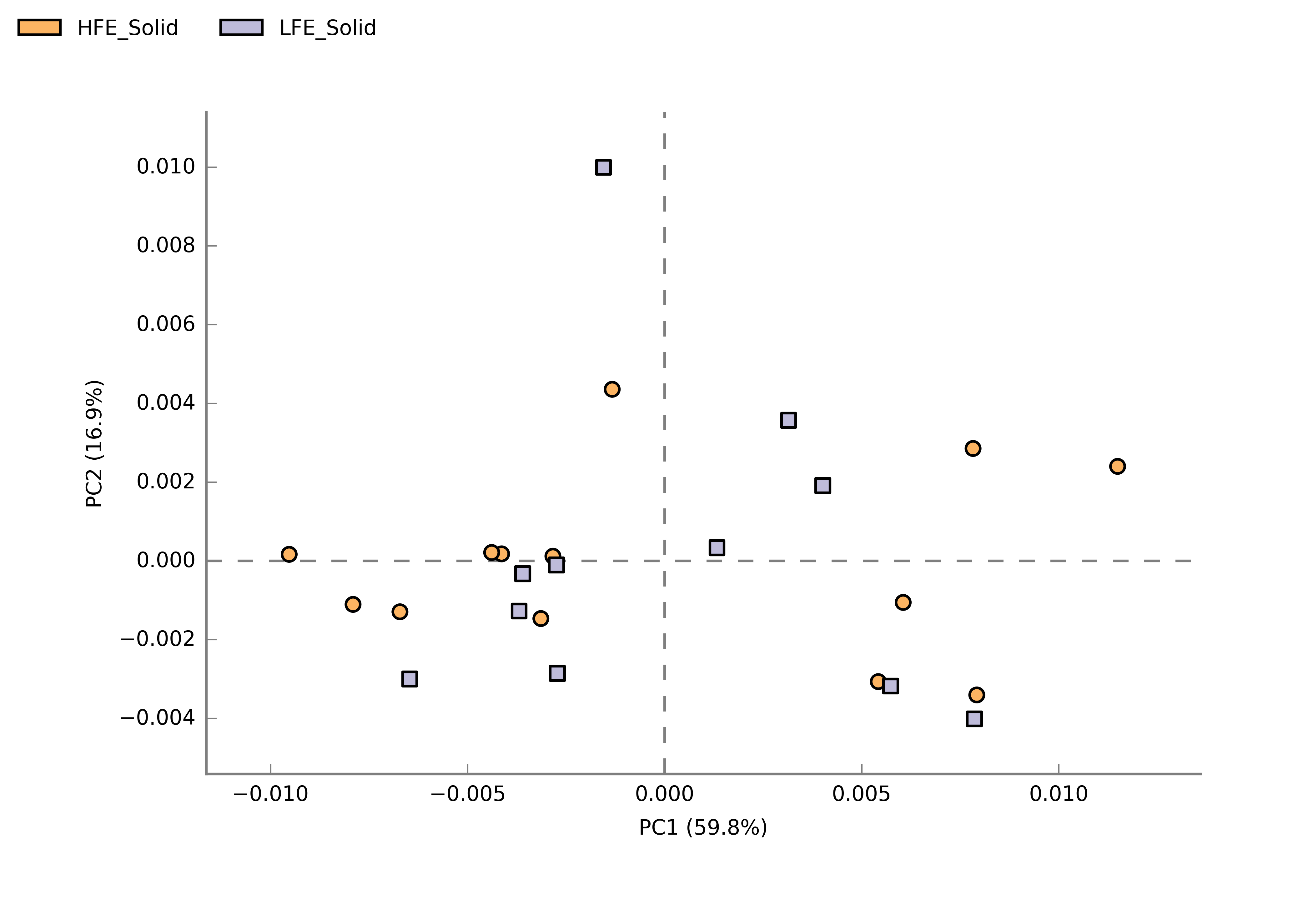

Supplement: FIGURE S5 — PCA plot comparing microbiome functional profiles for each sample sampled from the solid rumen phase. HFE n = 13, LFE n = 11. [file Image_5.JPEG]
